# Characterizing Antimicrobial Effects of Radiation and Antibiotic Interactions on *Staphylococcus aureus* and *Escherichia coli* Using MALDI-TOF MS

**DOI:** 10.3390/antibiotics14010041

**Published:** 2025-01-06

**Authors:** Ali Haider, Renáta Homlok, Csilla Mohácsiné Farkas, Tamás Kocsis

**Affiliations:** 1 Doctoral School of Food Sciences, Hungarian University of Agriculture and Life Sciences, H-1118 Budapest, Hungary; ali-haider90@hotmail.com; 2 HUN-REN Centre for Energy Research, H-1121 Budapest, Hungary; 3 Department of Food Microbiology, Hygiene, and Safety, Hungarian University of Agriculture and Life Sciences, H-1118 Budapest, Hungary; mohacsine.farkas.csilla@uni-mate.hu (C.M.F.); kocsis.tamas.jozsef@uni-mate.hu (T.K.)

**Keywords:** *Escherichia coli*, gamma irradiation, MALDI-TOF, piperacillin, protein, *Staphylococcus aureus*

## Abstract

**Background/Objectives:** Antibiotic-resistant bacteria are becoming a major challenge in human and veterinary medicine, as well as in food processing. **Methods:** In this study, the protein diversity in antibiotic-sensitive and -resistant strains of *Staphylococcus aureus* and *Escherichia coli* was investigated by exposing them to varying doses of gamma irradiation, with and without antibiotic presence. Changes in bacterial protein profiles were characterized using MALDI-TOF MS to reveal dose-dependent adaptations and potentiation effects under combined irradiation and antibiotic treatments. **Results:** The results indicate that MALDI-TOF MS effectively differentiates between sensitive and resistant strains, particularly at lower radiation doses (0, 0.2, and 0.4 kGy), with distinct separation in protein spectra. However, at 0.6 kGy, protein profiles plateaued, suggesting a potential threshold effect in radiation response. In 24-h cultures from irradiated *Staphylococcus aureus*, significant differences emerged in the resistant strain at 0.6 kGy in the presence of antibiotics, with further generational divergence dependent on initial antibiotic exposure. In the case of the sensitive strain, profiles were notably distinct at the 0.4 and 0.6 kGy doses, revealing dose- and treatment-specific responses. For *Escherichia coli*, generational differences between resistant and sensitive strains were apparent, though antibiotic effects on protein profiles were limited to the 0.6 kGy dose. **Conclusions:** The results underscore a potentiation interaction between irradiation and antibiotic exposure, affecting protein diversity and adaptation. Sensitive strains displayed heightened proteomic responses to minor treatment variations, while resistant strains exhibited more stable profiles across conditions. The findings highlight MALDI-TOF MS as a valuable tool in detecting proteomic biomarkers linked to bacterial resistance and stress adaptation.

## 1. Introduction

The global rise in antibiotic resistance poses a serious threat to public health, impacting clinical settings and environmental ecosystems. This challenge is further exacerbated by the presence of antibiotics and antibiotic-resistant bacteria (ARB) in natural water bodies, largely attributed to effluents from wastewater treatment plants (WWTPs) and agricultural runoff [1,2]. Antibiotic residues persist in aquatic environments, often bypassing conventional biological treatment processes in WWTPs, facilitating the selection and spread of resistant bacterial strains [3]. Therefore, addressing this issue requires interdisciplinary efforts involving engineers, microbiologists, and environmental scientists to develop and optimize new technologies capable of effectively degrading antibiotics and ARBs from these environments.

Antibiotics, such as β-lactams, have been widely used to treat a broad spectrum of bacterial infections. Still, their continued use has led to the emergence of resistant strains, notably methicillin-resistant *Staphylococcus aureus* (MRSA) [4]. The mecA gene, responsible for encoding penicillin-binding protein 2a (PBP2a), plays a crucial role in MRSA’s resistance mechanism by reducing its affinity for β-lactam antibiotics and other commonly used antibiotics, thus rendering treatments ineffective [5,6]. Horizontal gene transfer further contributes to the spread of resistance genes, amplifying the public health risk posed by these pathogens in clinical and environmental contexts [7,8,9,10].

In addition to clinical settings, WWTP effluents have been identified as critical control points in the environmental dissemination of antibiotic resistance [3]. Wastewater contains ARB and residual antibiotics, which serve as selective pressures for the development and spread of resistant genes in downstream water systems. Traditional treatment processes often fail to eliminate these contaminants, making advanced technologies essential for mitigating the impact of antibiotic resistance on the environment [2,11,12,13].

Among the emerging technologies, advanced oxidation processes (AOPs) show great potential for degrading antibiotics and removing ARBs from wastewater. AOPs, such as Fenton reactions, UV irradiation, and high-energy electron beam (EB) treatments, rely on the generation of highly reactive species like hydroxyl radicals (•OH) to oxidize and break down organic pollutants [14]. EB treatment, in particular, has gained attention due to its ability to generate both oxidizing (•OH) and reducing (eaq−) radicals without the need for chemical additives, making it a promising solution for large-scale wastewater applications [15]. Studies have demonstrated the effectiveness of EB treatment in degrading various pollutants, including antibiotics while reducing the antimicrobial activity of treated wastewater effluents [2,15,16,17,18,19,20,21,22,23].

The first attempts to identify microorganisms using mass spectrometry were performed as early as 1975 [24]. However, these experiments suffered from irreproducible results due to the variabilities caused by growth conditions and media. Only with the invention of matrix-assisted laser desorption/ionization time-of-flight mass spectrometry (MALDI-TOF MS) in the 1980s did the analysis of relatively large biomolecules, including larger ribosomal proteins, become possible [25]. Culture conditions influence the latter less, allowing MALDI-TOF MS to be consistently used to differentiate bacterial species [26,27].

MALDI-TOF MS has been employed to identify various microorganisms, such as bacteria, fungi, and viruses. Its ability to quickly and efficiently characterize microorganisms makes it highly promising for multiple applications, including medical diagnostics, biodefense, environmental surveillance, and food safety monitoring. This technology is well-suited for high-throughput microbial identification, offering rapid results at a lower cost and making it a viable alternative to traditional biochemical and molecular identification methods used in laboratories [28].

Several studies investigated the possibility of applying MALDI-TOF MS technology to the rapid detection of antibiotic resistance in bacterial pathogens isolated from bloodstream infections and the detection of antimicrobial resistance in pathogenic fungi [29].

MALDI-TOF MS has emerged as a highly sensitive analytical technique requiring minimal microbial biomass, typically ranging from 10⁴ to 10⁶ CFU for bacterial identification. The method employs a straightforward sample preparation process, where a small microbial sample is treated with formic acid and overlaid with a matrix solution containing α-cyano-4-hydroxycinnamic acid (α-HCCA) in a solvent mixture of acetonitrile and trifluoroacetic acid [30]. Upon drying, the sample and matrix crystallize on the target plate, forming a stable composite suitable for analysis.

The prepared plate is inserted into the mass spectrometer, where it undergoes analysis under high-vacuum conditions essential for optimal performance. Following vacuum stabilization, the sample is subjected to short laser pulses, vaporizing the matrix and ionizing ribosomal proteins within the sample. These ionized proteins are accelerated by a strong electromagnetic field and travel through the instrument’s flight tube. The “time of flight” (TOF) is meticulously measured, as it is influenced by the proteins’ mass and charge, generating a characteristic spectrum unique to each microbial species [31].

This spectral data serves as a molecular fingerprint, enabling precise microbial identification by comparing the generated spectrum to reference profiles in a comprehensive database. While the spectra are influenced by method-inherent variability, the approach remains a robust and reliable tool for differentiating between microbial species. The increasing adoption of MALDI-TOF MS highlights its transformative potential in microbiology, offering rapid, accurate, and cost-effective diagnostics [32].In this study, we investigated the responses of two strains of *Staphylococcus aureus*—resistant (B.02174), sensitive (B.01755)—and *Escherichia coli*—resistant (B.02357), sensitive (B01748)—to gamma irradiation and the antibiotic piperacillin by MALDI-TOF MS. These strains were chosen based on findings from our previous works [24,25,26], where we examined their behaviors in mixed populations within a wastewater matrix, specifically focusing on their sensitivities to piperacillin. In this investigation, we opted to study each strain individually to provide clearer insights into their distinct responses to stressors. This approach allows us to understand better the mechanisms of antibiotic resistance, which is critical for informing strategies to combat this growing global health issue. Piperacillin, a clinically significant β-lactam antibiotic, plays a crucial role in the treatment of bacterial infections. Its widespread use and well-characterized susceptibility profile make it an ideal model compound for studies focusing on β-lactam antibiotics and their mechanisms of action. In our previous studies, we documented differences in susceptibility between the two strains, with the resistant strain (B.02174) exhibiting full resistance to both antibiotics. In contrast, the sensitive strain (B.01755) remained susceptible. This contrast provides a valuable framework for understanding how each strain responds to the combined stress factors of gamma irradiation and trace antibiotic exposure. By applying these combined stressors, we aimed to gain insights into the adaptive mechanisms underlying antibiotic resistance and how bacterial populations react to the interplay of gamma irradiation and low antibiotic concentrations. This knowledge is essential for developing targeted strategies to combat β-lactam resistance mechanisms, optimize the clinical use of piperacillin, and enhance treatment outcomes in bacterial infections.

To characterize the selected strains, we performed MALDI-TOF mass spectrometry analysis. This technique confirmed the phenotypic and proteomic differences between the resistant and sensitive strains. MALDI-TOF is advantageous for its rapid and precise identification capabilities and its ability to reveal subtle changes in protein expression profiles. Understanding these profiles is crucial for elucidating how different strains respond to environmental stressors, including antibiotic pressure.

## 2. Results

**Figure 1:**
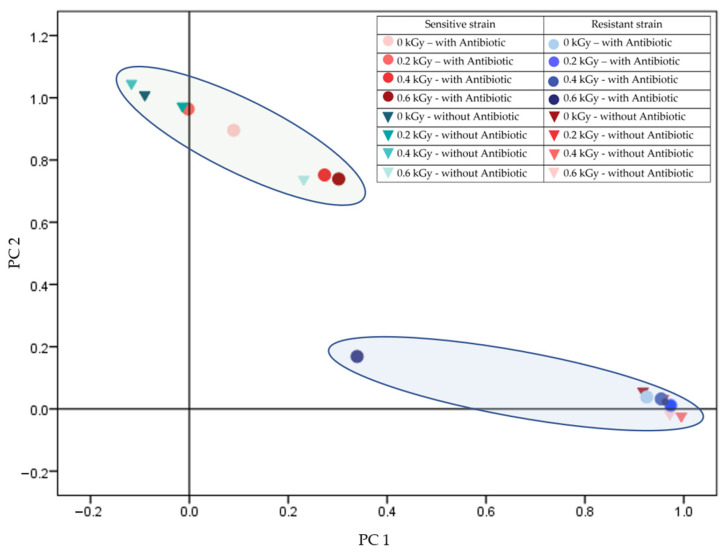
Comparison of resistant (B.02174) and sensitive (B.01755) *Staphylococcus aureus* in the presence of piperacillin (total variance: 93.505%; PC1: 83.622%; PC2: 9.882%).

Protein diversity arises primarily from variations in their amino acid sequences and post-translational modifications, such as phosphorylation and glycosylation, which influence their structure and function. MALDI-TOF MS (Matrix-Assisted Laser Desorption/Ionization Time-of-Flight Mass Spectrometry) has proven to be a valuable tool for detecting and characterizing diversity by enabling the measurement of molecular mass patterns. This technique can distinguish irradiated samples based on protein profiles, detect minor sequence variations, and analyze complex mixtures. It is particularly useful in proteomic studies that differentiate closely related proteins and identify specific biomarkers. Figure 1 shows the differentiation of protein profiles between sensitive and resistant *Staphylococcus aureus* strains at various radiation doses in the presence of piperacillin.

The protein spectra of the two strains showed a statistically significant separation when comparing Gram-positive *Staphylococcus* strains. In the case of the resistant strain, significant differentiation from the control was only observed at 0.6 kGy irradiation in the presence of the antibiotic. In all other treatments, the spectra did not separate significantly. Conversely, an irradiation dose effect was observed in the case of the antibiotic-sensitive strain. Trace amounts of the antibiotic did not significantly impact the protein patterns of the progeny cells in this case either (Figure 1).

**Figure 2:**
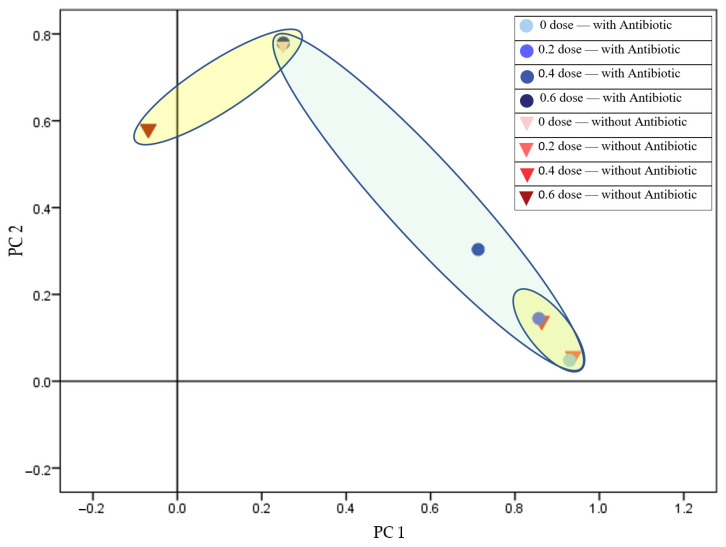
Separation of resistant *Staphylococcus aureus* (B.02174) based on radiation dose in the presence and absence of piperacillin (total variance: 97.160%; PC1: 93.991%; PC2: 3.169%).

When analyzing 24-h cultures derived from irradiated cells of the antibiotic-resistant strain (B.02174), a statistically significant effect of the irradiation dose was observed on the protein profiles of the bacterial populations. Conversely, the presence and varying concentrations of piperacillin, a β-lactam antibiotic, did not lead to any significant changes in the protein profiles (Figure 2).

**Figure 3:**
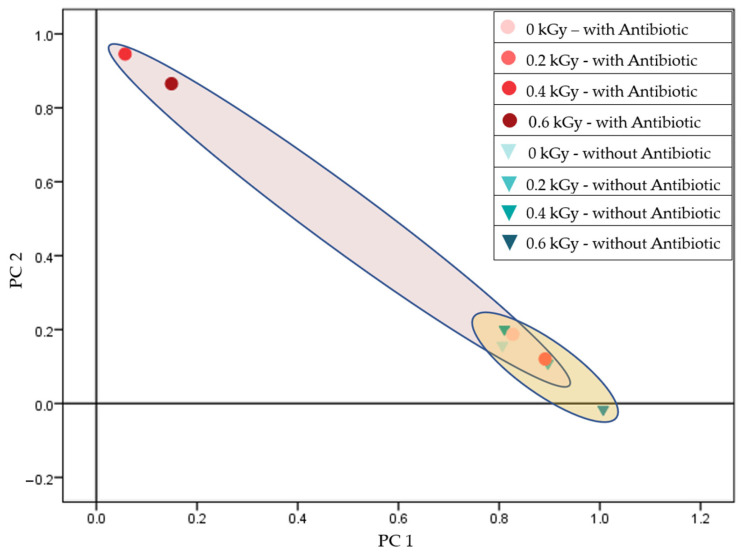
Separating sensitive *Staphylococcus aureus* (B.01755) based on radiation dose, in the presence and absence of piperacillin (total variance: 96.815%; PC1: 92.271%; PC2: 4.544%).

In examining the sensitive *Staphylococcus aureus* (B.01755) strain, the protein profiles synthesized from the 0.4 and 0.6 kGy treatments under antibiotic presence were significantly distinct from each other and from the other treatments. The protein profile at the 0.6 kGy dose was also differentiated from the control (Figure 3).

**Figure 4:**
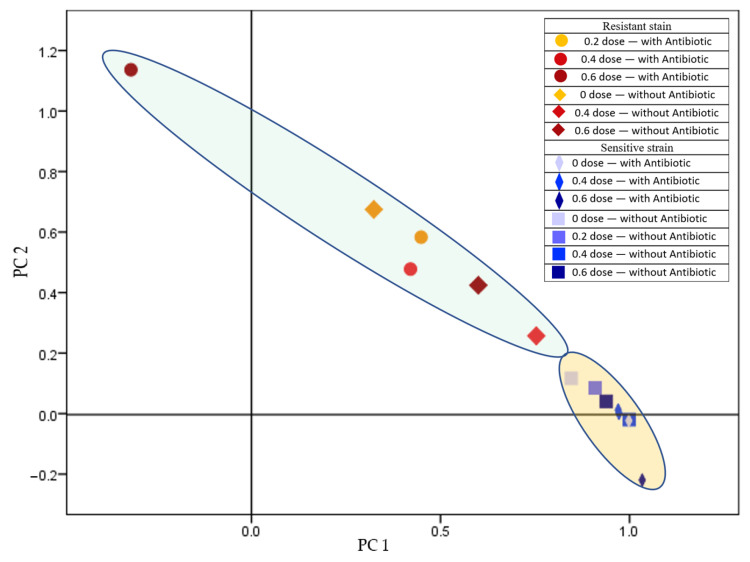
Comparison of resistant (B.02357) and sensitive (B01748) *Escherichia coli* stains in the presence and absence of piperacillin (total variance: 89.800%; PC1: 33.319%; PC2: 6.481%).

In a comparative analysis of the protein profiles of resistant and sensitive Gram-negative *Escherichia coli* strains in their subsequent generations, we found that the strains’ profiles were significantly distinct. The presence of antibiotics during the irradiation of the parental generations had a significant effect on the protein spectra of the progeny cells. An exception was observed at the 0.6 kGy dose, where irradiation in the presence of antibiotics resulted in significant differences from the control in both strains (Figure 4).

**Figure 5:**
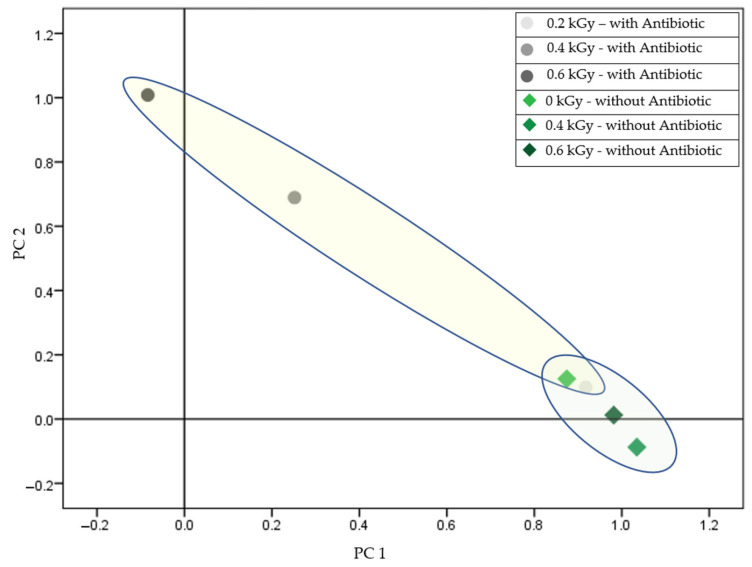
Separation of resistant *Escherichia coli* (B.02357) based on radiation dose, in the presence and absence of piperacillin (total variance: 92.138%; PC1: 82.330%; PC2: 9.808%).

When evaluating the data for the two strains separately based on the treatments, the resistant strain exhibited significantly different spectra in subsequent generations at the 0.4 and 0.6 kGy doses in the presence of antibiotics compared to the control. For the resistant strain, even a 0.4 kGy dose produced a lasting, statistically significant difference in the synthesized protein spectrum of progeny cells when trace amounts (2 µg/L) of piperacillin were present during irradiation (Figure 5).

**Figure 6:**
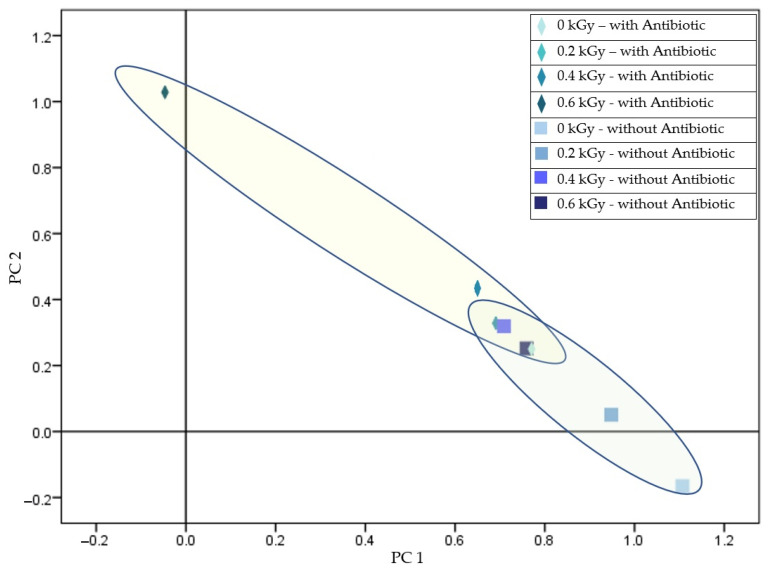
Separating sensitive *Escherichia coli* (B01748) based on radiation dose, in the presence and absence of piperacillin (total variance: 97.931%; PC1: 91.661%; PC2: 6.270%).

In contrast, for the sensitive strain, every treatment differed significantly from the untreated control, including the treatment containing trace amounts of antibiotics without irradiation. The potentiation effect of the two treatments, where their combined application resulted in greater antimicrobial efficacy than either treatment alone, is supported by the distinguishable groups based on dose effects. The treatment with antibiotics at the 0.6 kGy dose showed a significant distinction from the other treatments (Figure 6).

## 3. Discussion

The results demonstrate that MALDI-TOF MS effectively differentiated protein profiles between sensitive and resistant *Staphylococcus aureus* strains at 0, 0.2, and 0.4 kGy radiation doses, with clear dose-dependent distinctions. However, at 0.6 kGy, no significant differences were observed between strains, suggesting a potential threshold beyond which protein profile changes may plateau. Byrum et al. [33] have observed similar trends when investigating bacterial responses to radiation. Proteomic shifts stabilize at higher doses, indicating a saturation effect in cellular stress responses. The result depicted in Figure 2 highlights the differential radiation response of protein spectra, particularly in Gram-positive bacteria. Similar results were obtained using the PCR technique [34], which aligns with findings in prior studies utilizing genomic methods to analyze bacterial stress adaptations post-irradiation [35].

Further, in analyzing 24-h cultures derived from irradiated cells, a significant divergence was noted in the antibiotic-resistant *Staphylococcus aureus* strain at the 0.6 kGy dose compared to the control. Additionally, subsequent generations displayed pronounced differences in protein profiles depending on the presence or absence of antibiotics during initial irradiation, indicating a lasting effect on proteomic expression patterns in response to combined stressors (Figure 3). This observation is consistent with the results of studies demonstrating that combined stressors, such as antibiotics and radiation, lead to long-term proteomic and phenotypic adaptations in bacterial populations [36]. This is especially true when initial exposure conditions (e.g., the presence of antibiotics during irradiation) alter subsequent generations, emphasizing the long-term adaptability of bacterial populations in response to such challenges [37]. In the case of the sensitive strain, the 0.4 and 0.6 kGy treatments resulted in distinct profiles under antibiotic presence, differentiating these treatments not only from each other but also from other conditions, underscoring the interaction between radiation dose and antibiotic exposure in influencing protein expression (Figure 1). Prior research highlights similar synergistic effects between radiation-induced stress and antibiotic activity, particularly in Gram-positive bacteria, where radiation can weaken cell wall integrity, making bacteria more susceptible to antimicrobial agents [38,39].

In the case of Gram-negative *Escherichia coli* strains, resistant and sensitive strains exhibited significantly distinct protein profiles across subsequent generations, regardless of antibiotic presence during irradiation, except at 0.6 kGy. Antibiotic presence during irradiation introduced significant differences in protein profiles from controls in both strains (Figure 4). This suggests that, while radiation primarily influences protein diversity in these strains, the potentiation impact of antibiotics is notable only at higher radiation doses. Previous studies have shown that ionizing radiation disrupts bacterial protein expression, revealing vulnerabilities in cellular repair mechanisms that antibiotics can exploit, particularly in Gram-negative bacteria [40]. This combination is particularly relevant in addressing resistant bacterial populations, as the additional stress from irradiation may reduce bacterial adaptability and survival rates [41].

When analyzing treatments in the case of the resistant *Escherichia coli* strain, significant differences emerged in the 0.4 and 0.6 kGy doses in the presence of antibiotics relative to controls, indicating dose-responsive protein adaptations (Figure 5). Conversely, for the sensitive strain, each treatment exhibited significant variation from the untreated control, reinforcing the pronounced potentiation effect of combined irradiation and antibiotics (Figure 6). This result highlights the heightened sensitivity of the strain’s proteome to even minimal treatment variations, supporting the hypothesis that, in certain cases, antibiotic exposure exacerbates the radiation effect. These findings align with several reports that found that combined stressors could trigger adaptive or degenerative responses in bacterial proteomes depending on strain sensitivity and environmental conditions [35,38,41]. Our results indicate that above a certain threshold (0.6 kGy), a potentiation effect emerges between the antimicrobial actions of the antibiotic and irradiation. No significant differences were observed in the total cell count between the untreated and irradiated (<0.6 kGy) cultures, and storage had no impact on this. The number of cultivable *Staphylococcus aureus* living cells decreased by four orders of magnitude, while the *Escherichia coli* count decreased by a minimum of three orders of magnitude when a 10⁹ CFU cm^−3^ suspension was irradiated with a dose of approximately 0.6 kGy. These results do not fully align with other studies on bacterial inactivation through ionizing radiation, where Gram-positive bacteria generally exhibit higher radiation resistance compared to Gram-negative strains [42].

In conclusion, MALDI-TOF MS provided an effective methodology for capturing subtle yet significant protein profile changes under irradiation and antibiotic conditions, especially in distinguishing between closely related strains. Our findings highlight the potential for MALDI-TOF MS to detect specific biomarkers linked to resistance and irradiation response, offering implications for therapeutic strategies targeting resistant bacterial populations and enhancing our understanding of protein adaptation mechanisms in microbial resistance. These results contribute to the perspective in which the MALDI-TOF MS is a robust tool for investigating stress-induced proteomic changes and identifying resistance biomarkers in clinically relevant bacterial strains [43,44].

## 4. Materials and Methods

In this study, we focused on *Staphylococcus aureus* and *Escherichia coli* as our test microorganisms due to their clinical relevance and rapid antibiotic resistance acquisition. We selected one resistant strain (B.02174) and one sensitive strain (B.01755) in the case of *Staphylococcus aureus*, and one resistant strain (B.02357) and one sensitive strain (B.01748) in the case of *E. coli*, based on findings from our previous work [24,25,26]. Strain B.02174 (*S. aureus*) is methicillin-resistant (*mecA*-positive), while B.01755 (*S. aureus*) is methicillin-sensitive (*mecA*-negative). Strain B.02357 (*E. coli*) exhibits extended-spectrum β-lactamase (ESBL) production, conferring resistance to β-lactam antibiotics, whereas B.01748 (*E. coli*) is non-ESBL-producing and susceptible to common antibiotics. These resistance profiles, determined using the disc diffusion method, are detailed in Table 1. The disc diffusion test was conducted following the recommendations of the European Committee on Antimicrobial Susceptibility Testing (EUCAST) and the guidelines provided by the United States Clinical and Laboratory Standards Institute (CLSI) [45]. This method, widely used in hospital laboratories to identify effective antibiotics for specific bacterial infections, involves measuring the inhibition zones around antibiotic discs as an indicator of antimicrobial activity. The results were evaluated using the EUCAST Breakpoint Table v. 6.0 (2016). This selection allows us to investigate the contrasting responses of a resistant and a sensitive phenotype, thereby providing valuable insights into the mechanisms of antibiotic resistance development and survival under stress conditions. A bacterial suspension was prepared from an overnight culture of *Staphylococcus aureus*, incubated at 37 °C to reach a density of 0.5 McFarland standard (1–2 × 10^8^ CFU/mL). The turbidity of the suspension was adjusted using a photometric device (McFarland Densitometer DEN-1B, Biosan, Riga, Latvia) to ensure consistency across samples.

Samples were irradiated in a panoramic SLL-01 60 Co γ-irradiation facility with 1.9 PBq activity. The dose rate, determined with ethanol–chlorobenzene dosimetry, was 2 kGy h^−1^, and the absorbed doses were as follows: 0.2, 0.4, and 0.6 kGy. For the irradiation, a specialized sample holder was designed to ensure consistent positioning and exposure of the bacterial suspensions. The samples were arranged vertically within the holder to minimize variability in dose absorption. Control samples, which were not subjected to gamma irradiation, were always kept in the same location as the treated samples except during the irradiation process. This ensured that the control samples underwent identical handling and environmental conditions as the irradiated samples, apart from the exposure to gamma rays. During the irradiation process, the ambient temperature in the chamber was maintained at 37 °C. The 2 kGy h^−1^ dose rate was chosen deliberately, as it was sufficiently distant from the radiation source to avoid significant thermal effects on the samples. This arrangement ensured that the stress responses observed were due to the irradiation itself rather than secondary heat effects. For the irradiation, 15 mL centrifuge tubes were utilized, containing 5 mL of the prepared bacterial suspension. The irradiation process aimed to expose the bacterial strains to varying doses of gamma rays to evaluate their responses to the stress imposed by irradiation.

In the samples that included antibiotics, trace amounts of piperacillin were introduced at concentrations of 2 µg/L. These concentrations were selected to simulate environmental levels and assess the impact of sub-inhibitory antibiotic exposure on bacterial survival and behavior. Antibiotics were added to the bacterial suspensions immediately after their preparation from 24-h cultures. The cell count was adjusted to a density of 0.5 McFarland standard (10⁸ cells/mL) using a DEN-1B Densitometer at a wavelength of 565 nm. The prepared suspensions were subsequently subjected to gamma irradiation. Following irradiation, the samples were plated on CASO agar plates and incubated at 37 °C for 24 h to allow bacterial colonies to grow. The grown colonies were then harvested and prepared for MALDI-TOF MS analysis, providing insights into proteomic changes induced by the combined stressors of gamma irradiation and antibiotic exposure. This methodology allowed for a detailed exploration of bacterial responses to trace antibiotics in conjunction with irradiation, shedding light on potential mechanisms of resistance and adaptation under environmentally relevant conditions.

The following consumables were used during the study: piperacillin sodium salt (CAS No. 59703-84-3) was obtained from Sigma Aldrich (Saint Louis, MO, USA); in the microbiological experiments, sodium chloride (Cat. No. 1.06404.1000), peptone (Cat. No. 1.11931.1000), bacteriological agar (Cat. No. 1.01615.1000), yeast extract (Cat. No. 1.11926.1000), and glucose (Cat. No. 1.08346.9029) were from Merck (Boston, MA, USA); and trypto-casein soy broth (CASO, product BK046HA) was purchased from Biokar Diagnostics (Allonne, France).

Following gamma irradiation, bacterial strains were plated on CASO agar and incubated for 24 h. Colonies were then analyzed using an extended direct transfer procedure. Each colony was placed on a Bruker ground steel target plate, treated with 1 µL of 70% formic acid, and, once air-dried, overlaid with 1 µL of α-cyano-4-hydroxycinnamic acid (HCCA) matrix, with the entire procedure carried out under laminar flow conditions. Mass spectra were recorded within the 2000–21,000 Da range to examine phenotypic and proteomic differences between the strains. The recorded *m*/*z* range from 2000 Da to 20,000 Da was divided into 3600 bins of 5 Da widths, which is close to the mass accuracy assumed by the database-matching algorithm (0.08%).

Spectra acquisition was performed using a Microflex LT/SH MALDI-TOF mass spectrometer (Bruker Daltonics GmbH & Co., Bremen, Germany) equipped with a nitrogen laser (lambda = 337 nm) operating at a frequency of 60 Hz in linear positive ion detection mode. 240 spectra were generated per sample using 40 laser-shot steps from random positions on each isolate. Calibration was conducted using the *E. coli* ribosomal protein standard. The system was controlled by MALDI Biotyper 3.0 and FlexControl 3.4 software, with FlexAnalysis handling baseline subtraction, smoothing, and peak picking.

The mass spectra (approximately 1300 data points per sample) were relative intensity normalized, and Principal Component Analysis (PCA) was applied to reduce dimensionality and identify the major sources of variance driving dataset variability. To determine the endpoint or cut-off values, we considered the variability observed in the data. The cut-off values were set to exclude data points below 2000 Da and above 21,000 Da. The homogeneity of variance was assessed using Levene’s test, and principal component analysis was performed at a 95% confidence level (*p* < 0.05). PCA is a multivariate technique used to reduce dimensionality when redundancy exists within the data. It creates a new coordinate system by forming linear combinations of the original coordinates (*m*/*z* values), resulting in principal components (PCs). This transformation repositions the origin to the center of mass and rotates the axes to align with the principal axes of the data distribution. The PCs are ordered based on the proportion of variance in the original dataset that they explain. Results were visualized using biplots, with treatment means highlighted to emphasize effects. Data were analyzed using IBM SPSS (v20) and MS Excel.

## 5. Conclusions

This study highlights key findings on protein diversity and bacterial responses to combined irradiation and antibiotic treatments. The distinct protein profiles of sensitive and resistant *Staphylococcus aureus* and *Escherichia coli* strains demonstrate that MALDI-TOF MS can differentiate bacterial responses to stressors. Dose-dependent changes in protein expression were observed, with higher radiation levels revealing clearer distinctions, while no threshold effects were observed at lower (<0.4 kGy) doses. Antibiotic exposure during irradiation caused significant shifts in the mass spectra, particularly in sensitive strains, suggesting potentiation and heightened vulnerability. The combined effects emphasize the complex interaction between irradiation and antibiotics, with implications for bacterial persistence, particularly in sewage treatment. MALDI-TOF MS proves valuable for identifying proteomic adaptations, offering the potential for targeting resistant bacterial populations and developing treatments based on protein vulnerabilities.

## Figures and Tables

**Table 1:** Resistance profiles of *Staphylococcus aureus* and *Escherichia coli* strains.

No.	Tested Antibiotic	Mean (mm)	Indication	Mean (mm)	Indication	Reference		
**1**	**Piperacillin 100 µg**	***S. aureus B.01755***		***S. aureus B.01755***		**Resistant**	**Intermediate**	**Susceptible**
		34.3	susceptible	11.7	resistant	– – –	– – –	>18–21
		***E. coli B.01748***		***E. coli B.02357***				
		30.0	susceptible	10.0	resistant			

## Data Availability

The raw data supporting the conclusions of this article will be made available by the authors upon request.

## References

[1] Bush K., Courvalin P., Dantas G., Davies J., Eisenstein B., Huovinen P., Jacoby G.A., Kishony R., Kreiswirth B.N., Kutter E. (2011). Tackling antibiotic resistance. Nat. Rev. Microbiol..

[2] Berendonk T.U., Manaia C.M., Merlin C., Fatta-Kassinos D., Cytryn E., Walsh F., Bürgmann H., Huovinen P., Stefani S., Schwartz T. (2015). Tackling antibiotic resistance: The environmental framework. Nat. Rev. Microbiol..

[3] Baquero F., Martínez J.-L., Cantón R. (2008). Antibiotic resistance in the environment: The role of environmental pollution and antibiotic misuse. Curr. Opin. Biotechnol..

[4] Wielders C.L.W., Fluit A., Brisse S., Verhoef J., Schmitz F.J. (2002). mecA gene is widely disseminated in *Staphylococcus* population. J. Clin. Microbiol..

[5] Bungau S., Tit D.M., Behl T., Aleya L., Zaha D.C. (2021). Aspects of excessive antibiotic consumption and environmental influences correlated with the occurrence of resistance to antimicrobial agents. Curr. Opin. Environ. Sci. Health.

[6] Homlok R., Kiskó G., Kovács A., Tóth T., Takács E., Farkas C.M., Wojnárovits L., Szabó L. (2021). Antibiotics in a wastewater matrix at environmentally relevant concentrations affect coexisting resistant/sensitive bacterial cultures with profound impact on advanced oxidation treatment. Sci. Total Environ..

[7] Centers for Disease Control and Prevention (CDC), U.S. Department of Health and Human Services (2019). Antibiotic Resistance Threats in the United States 2019. https://ndc.services.cdc.gov/wp-content/uploads/Antibiotic-Resistance-Threats-in-the-United-States-2019.pdf.

[8] Davies J., Davies D. (2010). Origins and evolution of antibiotic resistance. Microbiol. Mol. Biol. Rev..

[9] Lowy F.D. (2003). Antimicrobial resistance: The example of *Staphylococcus aureus*. J. Clin. Investig..

[10] World Health Organization (WHO) (2015). Global Action Plan on Antimicrobial Resistance. https://www.who.int/publications/i/item/9789241509763.

[11] Priyanka U., Nandan A. (2014). A review on antimicrobial resistance and its implications. Int. J. OHSFE-Allied Sci..

[12] Martinez J.L. (2009). Environmental pollution by antibiotics and its effects on the human microbiota. Environ. Pollut..

[13] Andersson D.I., Hughes D. (2014). Microbiological effects of antibiotic resistance. Nat. Rev. Microbiol..

[14] Buxton G.V., Spotheim-Maurizot M., Mostafavi M., Douki T., Belloni J. (2008). An Overview of the Radiation Chemistry of Liquids. Radiation Chemistry: From Basics to Applications in Material and Life Sciences.

[15] IAEA (2007). Radiation Processing, Environmental Applications.

[16] Nickelsen M.G., Cooper W.J., Lin K., Kurucz C.N., Waite T.D. (1994). Photodegradation of 2,4-Dichlorophenoxyacetic Acid in Water. Water Res..

[17] Bae B.-U., Jung E.-S., Kim Y.-R., Shin H.-S. (1999). Removal of Antibiotics in Aqueous Solutions by Ozone Treatment. Water Res..

[18] Tobien T., Cooper W.J., Nickelsen M.G., Pernas E., O’Shea K.E., Asmus K.-D. (2000). Photodegradation of Sulfamethoxazole in Water. Environ. Sci. Technol..

[19] Roshani B., Leitner N.K.V. (2011). Photocatalytic degradation of antibiotics in water. Chem. Eng. J..

[20] Abdou L.A.W., Hakeim O.A., Mahmoud M.S., El-Naggar A.M. (2011). Photocatalytic degradation of organic pollutants in wastewater. Chem. Eng. J..

[21] Kim T.H., Kim S.D., Kim H.Y., Lim S.J., Lee M., Yu S. (2012). Treatment of antibiotic-contaminated water by electrochemical oxidation. J. Hazard. Mater..

[22] Xu G., Yao J.-Z., Tang L., Yang X.-Y., Zheng M., Wang H., Wu M.-H. (2015). Degradation of antibiotics using advanced oxidation processes. Chem. Eng. J..

[23] Wang L., Batchelor B., Pillai S.D., Botlaguduru V.S.V. (2016). Removal of antibiotics from wastewater using membrane bioreactors. Chem. Eng. J..

[24] Anhalt J.P., Fenselau C. (1975). Identification of bacteria using mass spectrometry. Anal. Chem..

[25] Keys C.J., Dare D.J., Sutton H., Wells G., Lunt M., McKenna T., Shah H.N. (2004). Compilation of a MALDI-TOF mass spectral database for the rapid screening and characterisation of bacteria implicated in human infectious diseases. Infect. Genet. Evol..

[26] Jones J.J., Stump M.J., Fleming R.C., Lay J.O., Wilkins C.L. (2003). Investigation of MALDI-TOF and FT-MS Techniques for Analysis of *Escherichia coli* Whole Cells. Anal. Chem..

[27] Haider A., Ringer M., Kotroczó Z., Mohácsi-Farkas C., Kocsis T. (2023). The importance of protein fingerprints in bacterial identification: The MALDI-TOF technique. J. Environ. Geogr..

[28] Giebel R., Worden C., Rust S.M., Kleinheinz G.T., Robbins M., Sandrin T.R. (2010). Microbial fingerprinting using matrix-assisted laser desorption ionization time-of-flight mass spectrometry (MALDI-TOF MS): Applications and challenges. Adv. Appl. Microbiol..

[29] Florio W., Baldeschi L., Rizzato C., Tavanti A., Ghelardi E., Lupetti A. (2020). Detection of antibiotic-resistance by MALDI-TOF mass spectrometry: An expanding area. Front. Cell. Infect. Microbiol..

[30] Bizzini A., Greub G. (2010). Matrix-assisted laser desorption ionization time-of-flight mass spectrometry, a revolution in clinical microbial identification. Clin. Microbiol. Infect..

[31] Carlsohn E., Nilsson C.L. (2007). Proteomic Techniques for Functional Identification of Bacterial Adhesins. Lectins.

[32] Croxatto A., Prod’hom G., Greub G. (2012). Applications of MALDI-TOF mass spectrometry in clinical diagnostic microbiology. FEMS Microbiol. Rev..

[33] Byrum S.D., Burdine M.S., Orr L., Mackintosh S.G., Authier S., Pouliot M., Tackett A.J. (2017). Time-and radiation-dose dependent changes in the plasma proteome after total body irradiation of non-human primates: Implications for biomarker selection. PLoS ONE.

[34] Kovács M., Wojnárovits L., Homlok R., Tegze A., Mohácsi-Farkas C., Takács E., Belák Á. (2024). Changes in the behavior of *Staphylococcus aureus* strains in the presence of oxacillin under the effect of gamma radiation. Environ. Pollut..

[35] Gao L., Zhou Z., Chen X., Zhang W., Lin M., Chen M. (2020). Comparative proteomics analysis reveals new features of the oxidative stress response in the polyextremophilic bacterium *Deinococcus radiodurans*. Microorganisms.

[36] Hahn V., Zühlke D., Winter H., Landskron A., Bernhardt J., Sievers S., Kolb J.F. (2024). Proteomic profiling of antibiotic-resistant Escherichia coli GW-AmxH19 isolated from hospital wastewater treated with physical plasma. Proteomics.

[37] Pérez-Llarena F.J., Bou G. (2016). Proteomics as a Tool for Studying Bacterial Virulence and Antimicrobial Resistance. Front. Microbiol..

[38] Wang J., Zhuan R., Chu L. (2019). The occurrence, distribution and degradation of antibiotics by ionizing radiation: An overview. Sci. Total Environ..

[39] Zhang M.Q., Zhang X.Y., Zhang H.C., Qiu H.B., Li Z.H., Xie D.H., Sheng G.P. (2024). Gamma-ray irradiation as an effective method for mitigating antibiotic resistant bacteria and antibiotic resistance genes in aquatic environments. J. Hazard. Mater..

[40] Wong O.Y., Yau V., Kang J., Glick D., Lindsay P., Le L.W., Giuliani M. (2018). Survival impact of cardiac dose following lung stereotactic body radiotherapy. Clin. Lung Cancer.

[41] Dawan J., Ahn J. (2022). Bacterial stress responses as potential targets in overcoming antibiotic resistance. Microorganisms.

[42] Shang W., Rao Y., Zheng Y., Yang Y., Hu Q., Hu Z., Rao X. (2019). β-Lactam antibiotics enhance the pathogenicity of methicillin-resistant *Staphylococcus aureus* via SarA-controlled lipoprotein-like cluster expression. MBio.

[43] Schott A.S., Behr J., Quinn J., Vogel R.F. (2016). MALDI-TOF mass spectrometry enables a comprehensive and fast analysis of dynamics and qualities of stress responses of *Lactobacillus paracasei* subsp. paracasei F19. PLoS ONE.

[44] Chac D., Kordahi M., Brettner L., Verma A., McCleary P., Crebs K., DePaolo R.W. (2020). Proteomic changes in bacteria caused by exposure to environmental conditions can be detected by Matrix-Assisted Laser Desorption/Ionization–Time of Flight (MALDI-ToF) Mass Spectrometry. bioRxiv.

[45] Matuschek E., Brown D.F.J., Kahlmeter G. (2014). Development of the EUCAST disk diffusion antimicrobial susceptibility testing method and its implementation in routine microbiology laboratories. Clin. Microbiol. Infec..

